# Evaluating Alleged Malpractice Cases Against Cardiologists and Cardiothoracic Surgeons in The Netherlands

**DOI:** 10.1093/icvts/ivag148

**Published:** 2026-05-14

**Authors:** Zaneta N Harlianto, Netanja I Harlianto

**Affiliations:** Faculty of Medicine, Anton de Kom Universiteit Suriname, Paramaribo, Suriname; Department of Radiology, University Medical Center Utrecht & Utrecht University, Utrecht, 3584CX, The Netherlands

**Keywords:** malpractice, cardiothoracic surgery, cardiology, medical errors, jurisprudence, quality improvement

## Abstract

This study aimed to analyse alleged malpractice cases and outcomes against cardiologists and cardiothoracic surgeons in the Netherlands. The Dutch Medical Disciplinary Database was searched for verdicts of alleged malpractice cases published between January 2010 and October 2025. Data were collected on case details and outcomes, and descriptive statistics were employed. A total of 219 cases were included (182 cases for cardiology and 37 cases for cardiothoracic surgery). Most cases were due to incorrect treatment/diagnosis for cardiologists and cardiothoracic surgeons (57% and 68%, respectively). Against cardiologists, 45/182 cases (25%) were judged as (partially) founded, for which 25 warnings, 17 reprimands, 1 temporary suspension, and 1 full practice prohibition were imposed. Against cardiothoracic surgeons, 11/37 cases (30%) were judged as (partially) founded, for which 10 warnings were imposed. We report trends and outcomes of alleged malpractice claims against cardiologists and cardiothoracic surgeons in the Netherlands. The cases discussed in this article offer valuable insights that can help enhance patient care.

## INTRODUCTION

The fields of cardiology and cardiothoracic surgery are regarded as high-risk specialties, with a higher number of malpractice claims compared to other specialties.^1^^,^^2^ This can be attributed to the frequency of (serious) adverse events, as well as the high-risk interventional procedures within the specialty.^3^ Before malpractice can be claimed, it must typically be proved by the court that a physician acted negligently. Negligence is defined as conduct that falls short of the minimal standard of ordinary care required by law to shield patients from an unreasonable risk of harm, without malice.^4^ The practice of “defensive medicine” is not uncommon to avoid medical malpractice, with physicians ordering more examinations or performing additional procedures.^5^

Whilst most studies focus on malpractice against cardiologists and cardiothoracic surgeons in the United States, studies in Europe focusing on malpractice claims against cardiothoracic surgeons are limited. In the Netherlands, jurisprudence is different from other medical systems, as the aim is not to compensate patients or to prosecute physicians.^6^ The purpose of the Dutch system is to safeguard the public interest, enhance the standard of healthcare, and defend patients’ individual rights.^6^ Elucidating the characteristics of alleged malpractice cases may help cardiologists and cardiothoracic surgeons avoid common sources of error and raise the standard of care. The purpose of this study was to report on the frequency and outcomes of alleged malpractice cases against cardiologists and cardiothoracic surgeons in the Netherlands.

## METHODS

### Data source

The online database of the Dutch Medical Disciplinary Court (https://tuchtrecht.overheid.nl/) was searched for malpractice cases against cardiothoracic surgeons and cardiologists between January 2010 and October 2025, as only cases with verdicts from 2010 onwards were made available online. A systematic search was performed with the terms: “cardio-thoracaal”, “cardiothoracaal”, “longchirurg”, “thoraxchirurg”, “hartchirurg”, and “cardioloog”. In the Netherlands, malpractice cases may be filed by patients, their immediate relatives, individuals involved in their care, employers and the hospital board of directors, or by the Health and Youth Care Inspectorate, a governmental institution overseeing public health in the Netherlands.^7^ Cases are usually handled by 2 legal experts and 3 medical experts. Each case data is made publicly available online and anonymized one day after the verdict.^7^

The following disciplinary measures can be imposed by the court: (1) warning; (2) reprimand; (3) monetary fine; (4) temporary suspension; (5) partial practice prohibition; (6) full practice prohibition.^7^ Measures restricting professional practice are published as standard. These are disclosed as a note in the BIG register, a publication in the Government Gazette and in one or more local daily or weekly newspapers, and placement on a list on the BIG register website.^7^ We extracted case specific data, the year of verdict, category of filed complaint, date of case filing and verdict, resident or medical specialist, lawyer present, disciplinary measure, and appeals filed.

### Data analysis

Data were provided separately for cardiologists and cardiothoracic surgeons and analysed using descriptive statistics. Numerical data were expressed as median, interquartile range (IQR), and categorical data as frequency (%). Statistics were performed using R Statistical Software version 4.5.2.

### Ethical statement

Institutional review board approval was not applicable to our study as we used online and anonymized data.

## RESULTS

### Total cases of alleged malpractice

**Figure 1.**
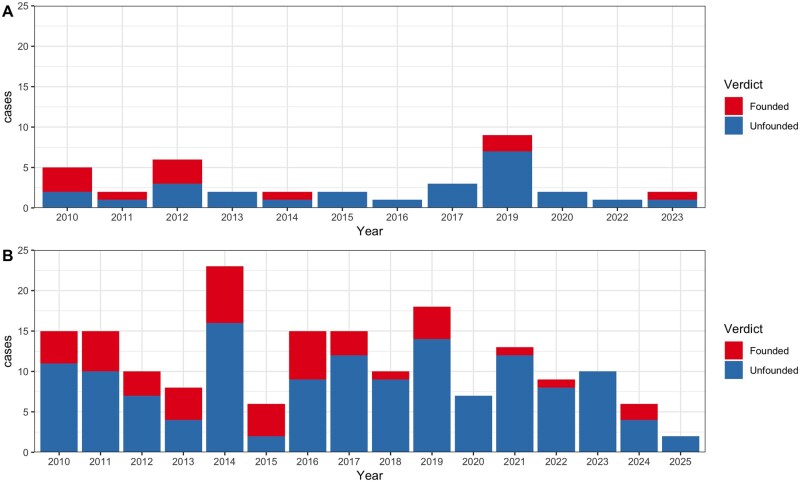
(A) Cardiothoracic Surgery Cases by Year. (B) Cardiology Cases by Year

A total of 219 cases of alleged malpractice were included, which were filed against cardiologists (*n* = 182, median 10 cases/year) and cardiothoracic surgeons (*n* = 37, median 2 cases/year) (Figure 1**)**.

### Allegations against cardiologists

For cardiology, 174 cases were filed against cardiologists and 8 against residents. Cases were filed by patients (*n* = 170), colleague physicians (*n* = 5), Health and Youth Care Inspectorate (*n* = 5), and hospital board of directors (*n* = 2). Alleged malpractice cases were most often related to incorrect treatment/diagnosis (*n *= 103; 57%), no/insufficient care (*n *= 43; 24%), insufficient information (*n *= 14; 8%), other (*n *= 11; 6%), incorrect statement/documentation (*n *= 6; 3%), no/delayed referral (*n *= 2; 1%), improper treatment (*n *= 3; 2%), and incorrect claim of costs (*n* = 1; 2%). The median time between case filing and verdict was 9.7 months (IQR: 7.1–12.6; range: 1.1–23.0). An attorney was present for most physicians (169/182; 93%), which was much lower for filing patients (70/170; 41%). The verdict was found to be (partially) founded in 45/182 cases (25%). Founded verdicts involved incorrect treatment/diagnosis (*n *= 29), no/insufficient care (*n *= 10), insufficient information (*n *= 2), incorrect statement/documentation (*n *= 3), and incorrect claim of costs (*n* = 1). The court imposed 25 warnings, 17 reprimands, 1 temporary suspension, 1 full practice prohibition, and no disciplinary measure in 1 case.

A qualitative description of verdict features is shown in Table. 1. A total of 64 appeals were filed by patients (*n* = 41), physicians (*n* = 13), physicians and patients (*n* = 6), Health and Youth Care Inspectorate (*n* = 2), Health and Youth Care Inspectorate and physician (*n* = 1), and hospital board of directors (*n* = 1). Following the appeals, 6 warnings and 2 reprimands were changed to unfounded, a warning was changed to a reprimand, and the full practice prohibition was changed to a partial practice prohibition.

**Table 1.** Qualitative Description of Certain Founded Verdicts

Selected cases cardiothoracic surgery
Only using chart review to decide that a patient who needs AVR, MVR, and TVR can wait at home. Thereafter, the patient’s condition deteriorated, and the patient passed away.
Not personally speaking with the patient before and after surgery; delegating the informed consent to a non-specialist doctor without ensuring the patient was adequately informed.
Failing to document in the patient file that the risks and possible complications of heart surgery were discussed with the patient.
The operative report was of poor quality, brief with multiple errors. Serious, irreversible damage occurred during surgery, including injury to the main bronchus and pulmonary artery. This was not reported to the inspection authority.
During AVR surgery, complications occurred, including an open pulmonary artery and left main bronchus, requiring a pneumonectomy. This was followed by right-sided heart failure, from which the patient died. A natural death was reported and communicated to the family.
The patient was incorrectly informed before surgery who would operate; During surgery, ventricular arrhythmias occurred and were incorrectly attributed to CO_2_ embolization. Further investigation was unjustifiably omitted. The patient deteriorated and died after surgery.
Performing rib resection and vertebral corpectomy, causing a permanent neurological deficit. No consultation took place with orthopaedic surgeons or with the bone tumour committee.
The surgeon had insufficient experience with tumour surgery involving the superior vena cava and the right lung lobe. Due to the slipping of a partial clamp, the patient died from brain damage.

Selected cases cardiology
Sending incorrect invoices to the patient.
ESC/EACTS guidelines recommended a 6-month follow-up for asymptomatic patients with severe aortic stenosis, but the patient’s condition had worsened on echocardiography, indicating the need for earlier reassessment, which was overlooked.
Failing to ensure proper follow-up of a patient with a prolonged QT interval on ECG. A first-degree relative of the patient died. Genetic testing showed that the patient had the genetic long QT syndrome.
Failing to recognize progression of hypertrophic cardiomyopathy. Necessary diagnostic tests were not performed, and echocardiograms showed progressive deterioration that was overlooked.
Patient with chest pain underwent a chest X-ray, showing abnormality requiring strict follow-up. Ten months later, the physician discovered the missed result, initiated follow-up imaging, and a lung adenocarcinoma with brain metastases was diagnosed. This oversight was incorrectly documented, and the error was not reported.
Misinterpreting exercise ECG: diagnosing a supraventricular tachycardia instead of bidirectional ventricular tachycardia. Failing to appreciate the severity of the findings and not initiating appropriate urgent management, such as admission, further diagnostics, or treatment, resulting in death.
Failing to diagnose Wolf-Parkinson-White on ECG, resulting in the death of a patient.
Not performing troponin investigations in a patient presenting with chest pain, resulting in a missed myocardial infarction and death.
Initially, treating a patient for acute coronary syndrome, who had symptoms that strongly suggested a possible pulmonary embolism. Not prioritizing an echocardiogram to rule out or confirm a large pulmonary embolism, given the clinical signs and risk factors.
Failing to review a radiology report on the day it became available and, when later reading it, missing that it described an aortic dissection, resulting in no timely operation and death.

### Allegations against cardiothoracic surgeons

A total of 37 cases were filed by patients between 2010 and 2025, against 36 cardiothoracic surgeons and 1 resident. These cases were classified as incorrect treatment/diagnosis (*n *= 25; 68%), no/insufficient care (*n *= 7; 18%), insufficient information (*n *= 2; 5%), other (*n *= 2; 5%), and improper treatment (*n *= 1; 3%). The median time between case filing and verdict was 9.6 months (IQR: 7.4–12; range: 0.5–32.3). Thirty-six out of 37 physicians had an attorney present (97%), which was lower for patients (60%). For 11/37 cases, allegations were classified as (partially) founded, for which 10 warnings and no disciplinary measures were imposed (incorrect treatment/diagnosis (*n *= 9) and no/insufficient care (*n *= 2)). Table 1 summarizes founded verdict cases. A total of 18 appeals were filed by patients (*n* = 13), physicians (*n* = 4), and both the patient and physician (*n* = 1). One unfounded case was changed to a warning after the appeal. The remaining appeals were rejected.

### Lessons to be learned from selected cases

(1) Obtaining informed consent is critical; (2) accurate documentation helps ensure continuity of care; (3) communication is important: discuss risks, benefits, and (treatment) alternatives; (4) ensure follow-up of abnormal results/findings; (5) consider multiple possible diagnoses; (6) adherence to guidelines and established clinical protocols; (7) communicate errors clearly and without concealment.

## DISCUSSION

To provide insight into malpractice in Europe, we report malpractice cases against cardiologists and cardiothoracic surgeons in the Netherlands. We found that between 2010 and 2025, 182 cases were filed related to cardiology (median 10 cases each year) and 37 cases against cardiothoracic surgeons (median 2 cases each year). Based on data from 2022, it is estimated that there were 1188 active cardiologists and 141 active cardiothoracic surgeons in the Netherlands,^8^ which corresponds to 0.96 yearly cases per 100 cardiologists and 1.7 yearly cases per 100 cardiothoracic surgeons.

Twenty-five percent in cardiology were (partially) founded, which was 30% for cardiothoracic surgery. Although disciplinary measures are done with the rationale to correct behaviour, it has been shown that these measures can have psychological effects and professional impact on physicians, including unhappiness, insecurity, and fear of additional malpractice lawsuits. In addition, negative factors included having a permanent record in the physician register, being viewed as guilty before the final verdict, online publication in newspapers, and the length of time for a verdict.^9^ Study participants who received a disciplinary measure reported feelings of being attacked, helplessness, anger, and being criminalized, and they were more likely to order more examinations (41%) or honour patient requests (35%).^10^ On the other hand, positive effects of these measures were also reported: more accurate documentation and discussing care improvement with co-workers.^10^ We provided examples of founded cases against cardiologists and cardiothoracic surgeons, where errors or misdiagnosis occurred.

Our findings are based on Dutch disciplinary law and are not generalizable to other countries with different judicial systems, which is a limitation of our study. The Dutch complaint system has as focus to protect the rights of the individual, whereas judicial procedures in other countries may have a larger focus on quality improvement and/or protecting the public interest.^6^ Another limitation is that we only included alleged malpractice cases, which is different from cases filed in civil court, where monetary compensation is claimed.

In conclusion, we report the trends and outcomes of alleged malpractice cases against cardiologists and cardiothoracic surgeons in the Netherlands. We provided examples of cases with founded verdicts, which included malpractice, medical errors, or misdiagnoses. The cases discussed in this article offer valuable insights that can help enhance patient care.

## Data Availability

Data are available from the corresponding author upon reasonable request.
